# Model selection for inferential models with high dimensional data: synthesis and graphical representation of multiple techniques

**DOI:** 10.1038/s41598-020-79317-8

**Published:** 2021-01-11

**Authors:** Eliana Lima, Robert Hyde, Martin Green

**Affiliations:** School of Veterinary Medicine and Science, University of Nottingham, Sutton Bonington Campus, Leicestershire, LE12 5RD UK

**Keywords:** Computational biology and bioinformatics, Risk factors, Mathematics and computing

## Abstract

Inferential research commonly involves identification of causal factors from within high dimensional data but selection of the ‘correct’ variables can be problematic. One specific problem is that results vary depending on statistical method employed and it has been argued that triangulation of multiple methods is advantageous to safely identify the correct, important variables. To date, no formal method of triangulation has been reported that incorporates both model stability and coefficient estimates; in this paper we develop an adaptable, straightforward method to achieve this. Six methods of variable selection were evaluated using simulated datasets of different dimensions with known underlying relationships. We used a bootstrap methodology to combine stability matrices across methods and estimate aggregated coefficient distributions. Novel graphical approaches provided a transparent route to visualise and compare results between methods. The proposed aggregated method provides a flexible route to formally triangulate results across any chosen number of variable selection methods and provides a combined result that incorporates uncertainty arising from between-method variability. In these simulated datasets, the combined method generally performed as well or better than the individual methods, with low error rates and clearer demarcation of the true causal variables than for the individual methods.

## Introduction

Inferential epidemiological research commonly involves identification of potentially causal factors from within high dimensional data spaces; examples include genetics, sensor-based data capture and large scale questionnaires. The selection of ‘important’ variables from within a high dimensional space is challenging because conventional stepwise selection procedures are known to perform poorly, resulting in inflated coefficients, downward biased errors and over fit models^1–4^. Over recent years, methods have been proposed in the statistical literature to improve variable selection for inference in high dimensional data, including modifications to AIC/BIC^5^, and a variety of regularisation methods based on functions that penalise model coefficients to balance over- and under-fitting (the variance-bias trade off)^6–8^. It has been shown, however, that different methods of variable selection can result in considerable differences in covariates selected^9^ and this poses difficult questions for the researcher about which method to choose, as well as presenting wider concerns around variability of results and therefore the reproducibility of science^10,11^.

To mitigate the issue of results being method-dependent, it has been argued that uncertainty in data are always explored from many angles^12^. Triangulation of multiple methods has been proposed as an aid to identify important variables^13^; in this context triangulation refers to conducting a variety of analytic methods on one set of data, on the premise that the most important variables will tend to be identified by most methods. Indeed, recent research has indicated this approach is likely to be beneficial^9^. However, rather than using triangulation to simply compare methods, a route to formally combine results from several statistical approaches would be advantageous to explicitly represent the additional uncertainty arising from variation between methods. Here we propose an approach to synthesise parameter estimates across different methods, to formally triangulate results and to compare, capture and account for between-method variability.

Furthermore, it is recognised that robustness in model selection can be improved through use of selection stability^14,15^. The concept is that covariates most frequently selected under repeated resampling are most likely to be of importance in a target population. Resampling, such as bootstrapping, is effective to evaluate selection stability^14^ and has the advantage of simultaneously providing an estimate of model coefficient distributions^4^, both of which can be used to provide a ranking of the relative importance of potential covariates^16^.

In this paper we propose an adaptable method to compare and synthesise results across any chosen number of variable selection techniques to triangulate results between methods. The method incorporates selection stability and coefficient distributions to provide an overall, unified result that encompasses the uncertainty arising between methods. To illustrate the method, we also describe a simple graphical technique to compare individual and synthesised models which readily allows identification and visualisation of the important variables selected by each, and the combination of methods.

## Results

### The data

Seven datasets were used to compare and synthesise results from six methods of automated variable selection. Six datasets were simulated such that underlying relationships were known a priori within the data and one dataset was from a previously conducted field study, to illustrate the proposed methods using real data. Construction of the simulated datasets is described in detail in the “Materials and Methods” section.

The simulated datasets used were of different dimensions. Datasets 1 and 2 were constructed by randomly simulating 1000 observations (rows) for 910 theoretical potential explanatory covariates (columns), ten of which were specified to have a known relationship with an outcome variable. Datasets 3 and 4 were constructed by randomly simulating 200 observations for 10,000 potential explanatory covariates, again only ten of which were specified to have a known relationship with an outcome variable. All covariates were simulated to have a mean = 0 and a standard deviation = 1 (i.e. they represented standardised variables) and each dataset allowed the outcome variable, “y_out”, to be calculated with a known degree of uncertainty. Datasets 5 and 6 comprised 1000 observations by 910 covariates, and 200 observations by 10,000 covariates respectively, but an outcome variable was generated at random (i.e. independent of and with no underlying relationship to, the potential explanatory covariates). The real dataset was gathered from a study conducted on 408 commercial sheep farms in the UK^16^.

### Results: datasets 1 and 2

The signal from the true variables was set to be stronger in Dataset 2 (the ten true variables explained 73% of variability in the outcome) than Dataset 1 (28% variability explained) but both datasets were considered to be representative of possible realistic field data. The partial coefficient distributions of the variables providing the ‘true’ underlying signal in these datasets are provided in Table 2.

Statistical methods implemented to conduct variable selection within these datasets were stepwise linear regression (SLR) based on AIC, elastic net regression (enet), smoothly clipped absolute deviation (SCAD), minimax convex penalty (MCP), SparseStep regression, and stepwise selection based on a modified Bayesian Information Criterion (mBIC). These statistical approaches were employed using standard methodology which is described in detail in the “Materials and Methods” section. Implementation of these six statistical approaches, without bootstrapping, resulted in different subsets of variables being selected using each method as described in Table 1. For both datasets, the false positive error rate was, as expected, substantially higher for the models using a conventional stepwise AIC method than for all other methods; this demonstrates the substantial over-fitting that occurs using this methodology with high dimensional data. For Dataset 1, elastic net, SCAD and MCP each selected a number of false positive variables (false positive error rate (FPER) 3.2 – 4.4%) but with a relatively low false negative error rate (elastic net and SCAD models contained no false negatives). In contrast, modified BIC and SparseStep models resulted in sparser models with low false positive rates (≤ 0.1%) but at the expense of omitting true variables, and hence a relatively high false negative rate (50–70%). For Dataset 2, in which a stronger signal was provided by the ten true variables, all models demonstrated reduced error rates. There were both fewer false positive and negative variables selected, although no method correctly allocated all variables. Despite the strong signal in the data, it was noticeable how poorly selection using conventional AIC performed (FPER = 15%).

**Table 1:** Error rates in variable selection of six statistical methods conducted on two simulated datasets.

	Model type					
	AIC	Enet	MCP	mBIC	SCAD	Sp_Step
**Dataset 1**						
Number false positives	234	36	29	0	40	1
False positive error rate	26.0%	4.0%	3.2%	0.0%	4.4%	0.1%
Number false negatives	1	0	1	7	0	5
False negative error rate	10.0%	0.0%	10.0%	70.0%	0.0%	50.0%
**Dataset 2**						
Number false positives	135	6	1	0	1	0
False positive error rate	15.0%	0.7%	0.1%	0.0%	0.1%	0.0%
Number false negatives	0	0	1	1	1	1
False negative error rate	0.0%	0.0%	10.0%	10.0%	10.0%	10.0%

Datasets 1 and 2 both contained 1000 observations and 910 potential explanatory covariates of which 10 were simulated to be ‘correct’ covariates and calculated to have a true effect on the outcome. The true covariates in Dataset 1 explained 28% of the variability in outcome and those in Dataset 2 explained 73%.

*AIC* Stepwise selection based on Akaike Information Criterion, *Enet* elastic net regression, *MCP* minimax convex penalty, *mBIC* modified Bayesian Information Criterion, *SCAD* smoothly clipped absolute deviation, *Sp_Step* SparseStep regression.

The coefficient estimates for the variables selected in these models are provided in Table 2. For Dataset 1, of the variables selected using each approach, elastic net, SCAD and MCP tended to shrink coefficients towards zero whereas modified BIC and SparseStep tended to slightly inflate coefficient estimates compared to the underlying true partial coefficients. The results illustrate the substantive variations in both variables selected and coefficient values dependent upon the method adopted. For Dataset 2 with the stronger signal, coefficient estimates tended to be more similar between methods and closer to the true central estimate for all methods, although the same general tendency for bias occurred for each method.

**Table 2:** Comparison of coefficient estimates for ten ‘true’ variables specified in the underlying model for Datasets 1 and 2 for six statistical methods of variable selection.

Model type									
Model term	True	True (95% CI)		AIC	Enet	MCP	mBIC	SCAD	Sp_Step
**Dataset 1**									
X1	3.23	(1.77	4.69)	4.25	3.23	4.28	4.40	4.56	4.78
X2	1.48	(0.11	2.84)	1.37	1.01	–	–	0.12	–
X3	2.39	(0.97	3.82)	1.69	1.95	2.87	3.05	3.14	–
X4	2.23	(0.86	3.60)	1.30	1.80	1.71	3.16	1.84	2.94
X5	1.79	(0.37	3.22)	2.39	1.66	1.77	–	0.79	2.91
X6	2.24	(1.26	3.21)	2.84	1.20	2.02	–	1.75	2.11
X7	1.98	(1.02	2.94)	1.38	0.89	1.30	–	1.09	2.18
X8	1.29	(0.32	2.27)	–	0.31	0.26	–	0.31	–
X9	2.28	(1.32	3.24)	1.68	1.20	1.99	–	1.77	–
X10	1.75	(0.79	2.72)	0.86	0.72	0.99	–	0.78	–
**Dataset 2**									
X1	2.39	(1.78	2.99)	2.11	2.10	2.62	2.63	2.62	2.63
X2	1.06	(0.48	1.64)	1.12	0.94	–	–	–	–
X3	2.66	(2.06	3.26)	2.33	2.49	2.91	2.92	2.91	2.92
X4	2.48	(1.91	3.05)	2.67	2.35	2.70	2.69	2.70	2.69
X5	2.88	(2.30	3.46)	3.07	2.88	3.09	3.09	3.10	3.09
X6	2.38	(1.97	2.80)	2.41	1.92	2.39	2.39	2.39	2.39
X7	2.58	(2.18	2.99)	2.44	2.10	2.61	2.61	2.61	2.61
X8	2.51	(2.10	2.91)	2.73	2.09	2.50	2.50	2. 49	2.50
X9	2.53	(2.10	2.95)	2.38	2.05	2.54	2.54	2.55	2.54
X10	2.23	(1.82	2.65)	2.11	1.75	2.21	2.22	2.21	2.22

Both datasets contained 1000 observations and 910 potential explanatory covariates.

*True* the correct partial coefficient distribution, *AIC* Stepwise selection based on Akaike Information Criterion, *Enet* elastic net regression, *MCP* minimax convex penalty, *mBIC* modified Bayesian Information Criterion, *SCAD* smoothly clipped absolute deviation, *Sp_Step* SparseStep regression.

### Multiple method comparisons and synthesis; datasets 1 and 2

**Figure 1:**
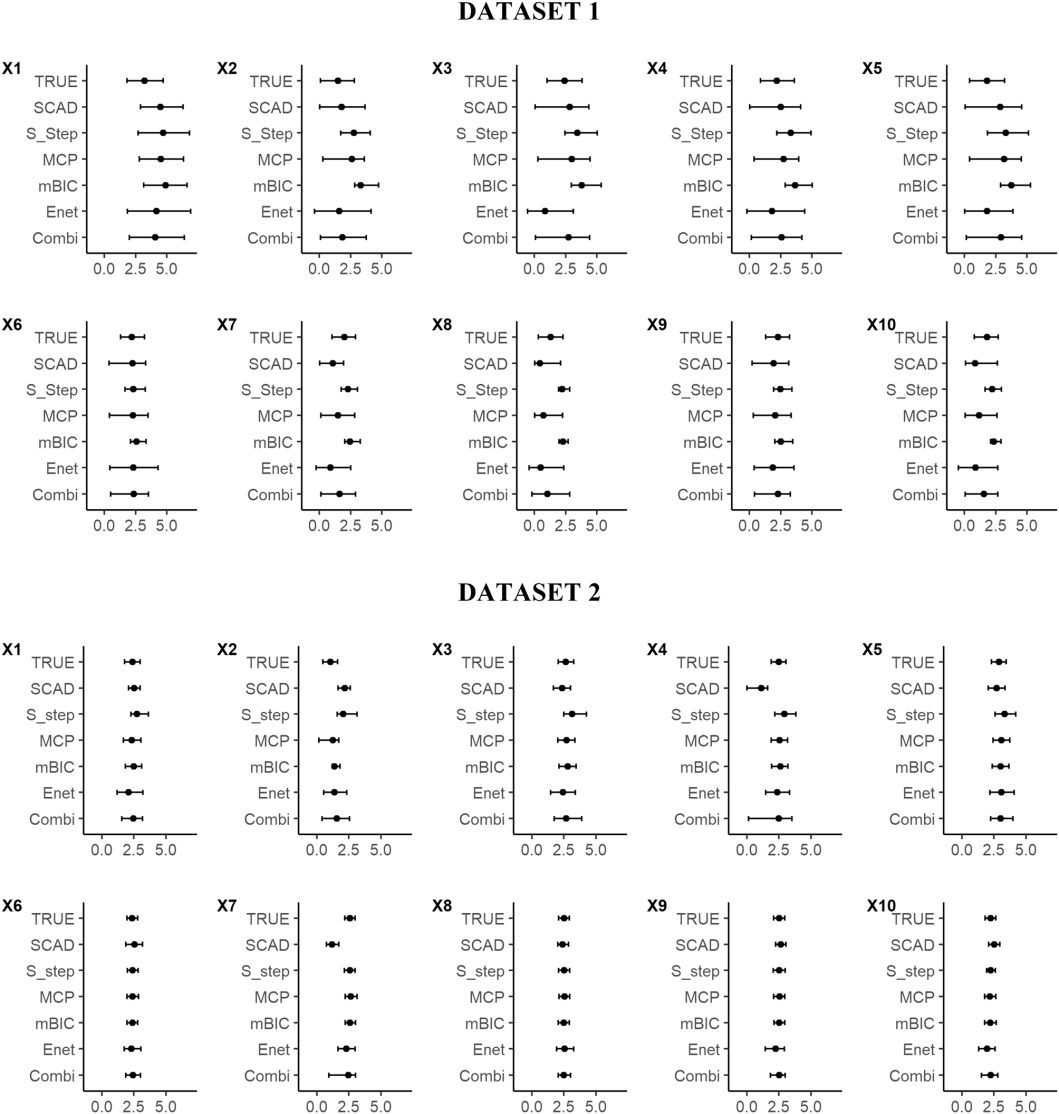
Bootstrap coefficient distributions of the true covariates in Datasets 1 and 2, estimated using five different statistical techniques and a combination method incorporating all five. ‘TRUE’ represents the actual underlying true partial coefficients. Key; X-axes – covariate coefficient value, X1 to X10 – covariate names, TRUE – the correct partial coefficient distributions for each covariate, SCAD – smoothly clipped absolute deviation, S_Step – SparseStep regression, MCP – minimax convex penalty, mBIC – modified Bayesian Information Criterion, Enet – elastic net regression, Combi – Combined method; results aggregated from all five techniques.

Covariate coefficients and selection stability were estimated for all models using a bootstrap methodology, except for the conventional SLR based on AIC which was deemed to perform too poorly to carry forward. The procedures for bootstrapping and calculating selection stability are described in detail in the “Materials and Methods” section. Model coefficient distributions derived from 500 bootstrap samples from Datasets 1 and 2, for each method, are illustrated in Fig. 1. For Dataset 1, in general the bootstrap intervals had reasonable overlap with the true covariate distribution, although mBIC and SparseStep had a tendency for overestimation and elastic net a slight tendency for underestimation. For Dataset 2, all estimated coefficient distributions were tighter reflecting the narrower true intervals from the stronger signal in these data. However, there remained variation in estimated coefficient distributions between method, especially for covariates X2, X4 and X7.

**Figure 2:**
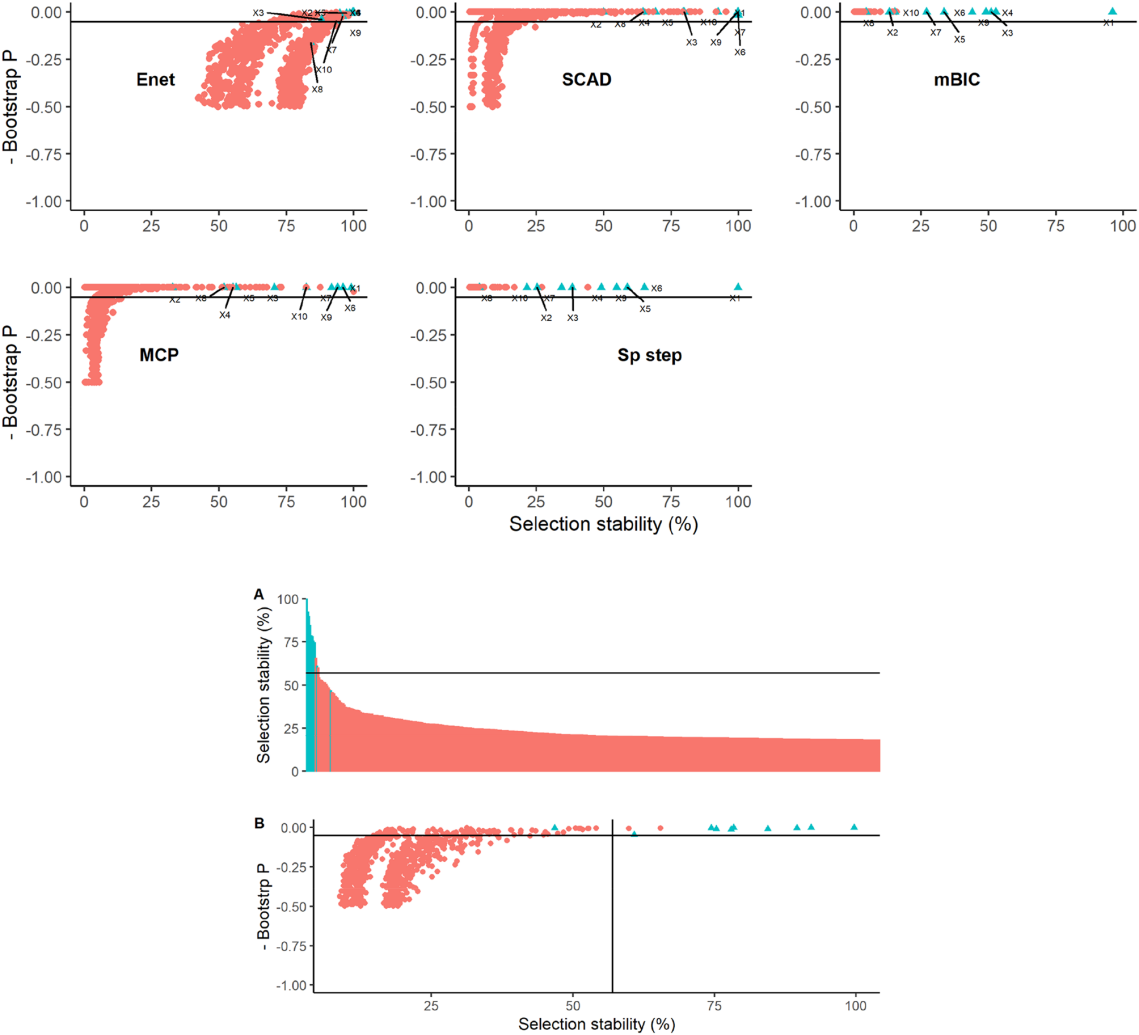
Graphical illustrations of bootstrap results from Dataset 1 using five methods of covariate selection. Graphs depict scatterplots of negative bootstrap P value against covariate selection stability except for Graph A that is an ordered plot of covariate selection stability in descending order for the combined model aggregating all five methods. The lines on graphs A and B represent the calculated threshold to determine a cut-off for ‘important’ covariates. The ‘true’ underlying covariates are coloured blue and labelled X1–X10.

Covariate selection stability and bootstrap P values estimated for Dataset 1, for each statistical method and all covariates, are illustrated in Fig. 2. The between method variability in selection stability is evident with elastic net, SCAD and MCP tending to select more variables more often (at > 50% stability) than SparseStep and mBIC. Although for each method, the true variables tended to be ranked relatively highly in terms of stability, there remained considerable overlap with false variables. This overlap was generally less with mBIC and Sparsestep although the actual stability values were lower with these methods. The true variables X1, X6 and X9 were generally relatively stable and with a low bootstrap P value in all methods, but clear demarcation and similar ranking of the remaining true variables was not evident across methods. The combined stability and bootstrap P values calculated by synthesising results across methods, are also displayed in Fig. 2. The combined method provides a formal approach for triangulation of results across methods by combining the bootstrap coefficient matrices for all methods to calculate an overall stability and bootstrap P value for each variable. The combined method provided a clearer separation of the true variables indicating that in general, across methods, the true variables tended to be selected most commonly by all methods and with low bootstrap P values. Using a rolling mean rate of change of selection stability > 1 to identify a threshold above which variables were deemed ‘important’ (Fig. 2), nine of the ten true variables would be selected as important (FNER = 10%) and two false variables would incorrectly be identified as positive (FPER = 0.2%); this compared favourably with the error rates of full models of the different methods presented in Table 1.

The coefficient distributions of the combined method are illustrated in Fig. 1. These distributions showed a good coverage of the true covariate distributions although were generally slightly conservative (wider) than those in the true underlying model. The combined distributions represent a non-parametric, weighted average across models and since they were estimated from covariates selected in each bootstrap sample, they were effectively weighted by selection stability of each method. These combined coefficients therefore represent the combined uncertainty in covariate estimates arising from variability between method.

**Figure 3:**
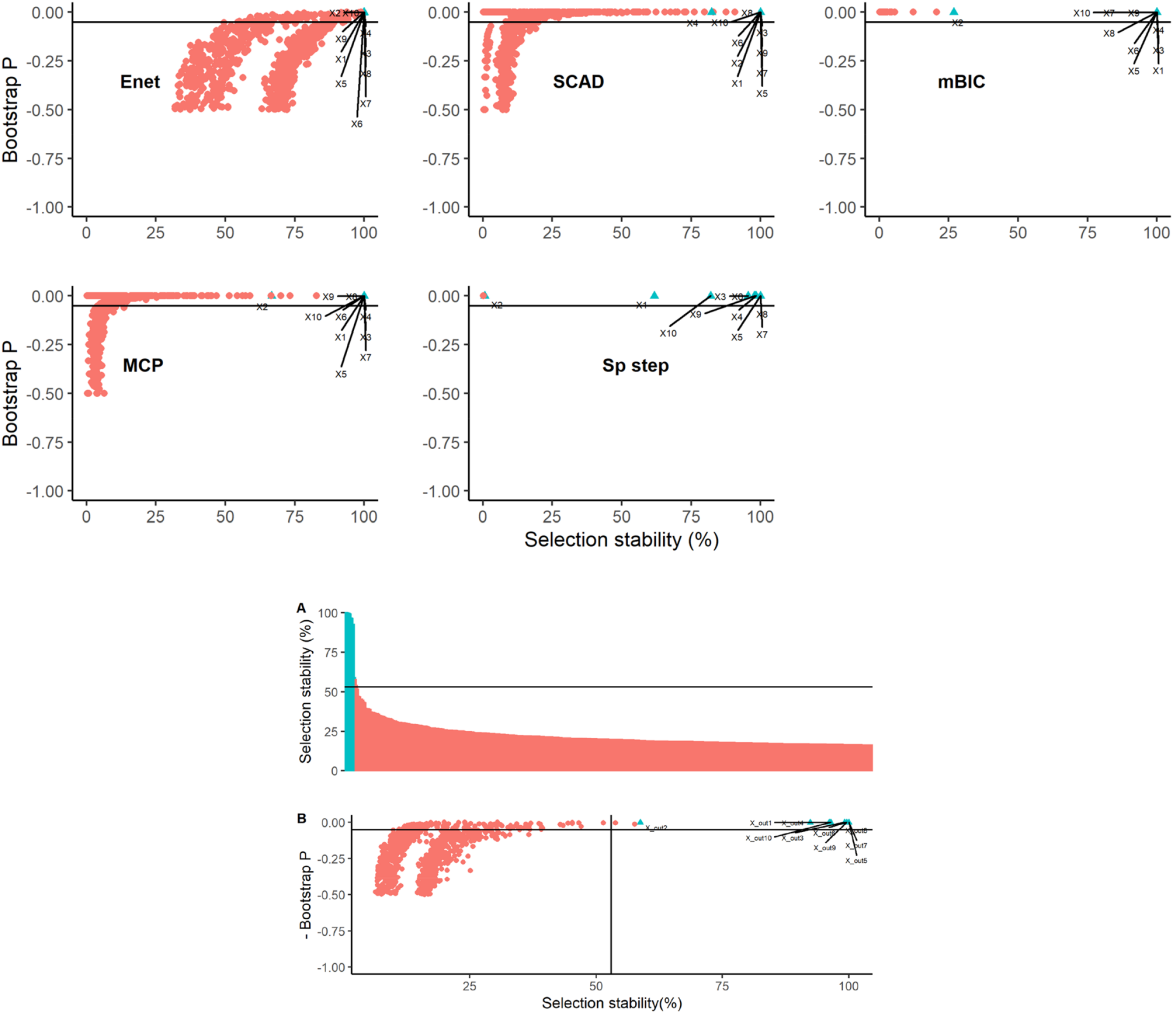
Graphical illustrations of bootstrap results from Dataset 2 using five methods of covariate selection. Graphs depict scatterplots of negative bootstrap P value against covariate selection stability except for Graph A that is an ordered plot of covariate selection stability in descending order for the combined model aggregating all five methods. The lines on graphs A and B represent the calculated threshold to determine a cut-off for ‘important’ covariates. The ‘true’ underlying covariates are coloured blue and labelled X1–X10.

Covariate selection stability and bootstrap P values estimated for Dataset 2 are illustrated in Fig. 3. With the stronger signal, all methods performed better than with Dataset 1 with the true variables generally being most stable and with low bootstrap P values. All methods had difficulty in differentiating true covariate X2 (the covariate with the smallest true effect size and true confidence interval closest to zero) and elastic net and SCAD tended to select false variables more commonly in bootstrap samples than other methods. The combined stability and bootstrap P values calculated across all methods are also displayed in Fig. 3. The combined method provided a clear separation of the true variables with the exception of variable X2 that was problematic for each method. Using the rolling mean rate of change of selection stability > 1 to identify a threshold above which variables were deemed ‘important’ (Fig. 3), all ten true variables were selected as important (FNER = 0%) and two false variables were incorrectly be identified as positive (FPER = 0.2%); this is similar to the error rates of the full models of different methods, presented in Table 1.

The coefficient distributions of the combined method for Dataset 2 are illustrated in Fig. 1. These distributions displayed good coverage of the true covariate distributions although, as with Dataset 1, were slightly conservative (wider) than those in the true underlying model. As for Dataset 1, the wider coefficient intervals from the combined method represents the uncertainty in covariate estimates arising from variability between methods.

### Results: datasets 3 and 4

The same analytic work flow was conducted on the two larger simulated datasets in which p (number of covariates) was much greater than n (number observations), (p = 10,010 n = 200). Dataset 3 had the stronger signal (ten true variables explained 73% of variability in the outcome) than Dataset 4 (63% variability explained). The partial coefficient distributions of the variables providing the ‘true’ signal in these datasets are illustrated in Fig. S1, in Supplementary Information. The FPER and FNER for each of the full models are provided in Table 3. In general, none of the methods performed well with these datasets; all models had a relatively high FNER (30–80%) with the exception of elastic net for Dataset 3 in which the FNER was zero but which selected 111 false positive variables (FPER = 1.1%).

**Table 3:** Error rates in variable selection of six statistical methods conducted on two simulated datasets.

	Model					
	AIC	Enet	MCP	mBIC	SCAD	Sp_Step
**Dataset 3**						
Number false positives	199	111	14	0	0	0
False positive error rate	1.99%	1.11%	0.14%	0.00%	0.00%	0.00%
Number false negatives	1	0	5	6	3	4
False negative error rate	10.0%	0.0%	50.0%	60.0%	30.0%	40.0%
**Dataset 4**						
Number false positives	200	6	3	0	2	78
False positive error rate	2.00%	0.06%	0.03%	0.00%	0.02	0.78%
Number false negatives	5	4	6	8	4	3
False negative error rate	50.0%	40.0%	60.0%	80.0%	40.0%	30.0%

Datasets 3 and 4 both contained 200 observations and 10,010 potential explanatory covariates of which 10 were simulated to be ‘correct’ covariates and calculated to have a true effect on the outcome. The true covariates in Dataset 3 explained 73% of the variability in outcome and those in Dataset 4 explained 63%.

*AIC* Stepwise selection based on Akaike Information Criterion, *Enet* elastic net regression, *MCP* minimax convex penalty, *mBIC* modified Bayesian Information Criterion, *SCAD* smoothly clipped absolute deviation, *Sp_Step* SparseStep regression.

### Multiple method comparisons and synthesis; datasets 3 and 4

Model coefficients distributions derived from the bootstrap sampling are illustrated in Fig. S1 in Supplementary Information. For both datasets there was substantial variability in estimated covariate distributions between method, with both the central location and 95% bootstrap intervals varying greatly.

**Figure 4:**
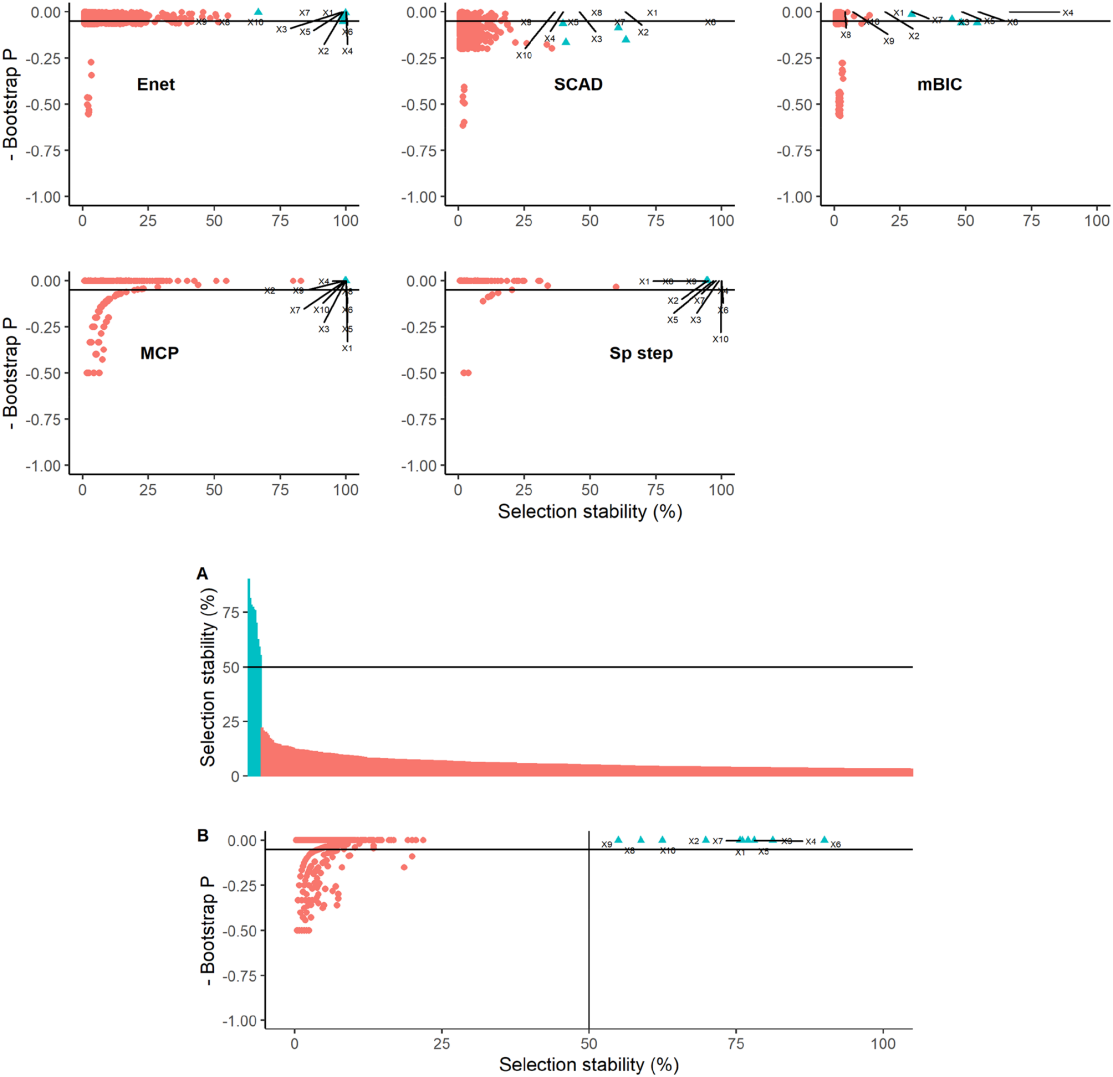
Graphical illustrations of bootstrap results from Dataset 3 using five methods of covariate selection. Graphs depict scatterplots of negative bootstrap P value against covariate selection stability except for Graph A that is an ordered plot of covariate selection stability in descending order for the combined model aggregating all five methods. The lines on graphs A and B represent the calculated threshold to determine a cut-off for ‘important’ covariates. The ‘true’ underlying covariates are coloured blue and labelled X1–X10.

**Figure 5:**
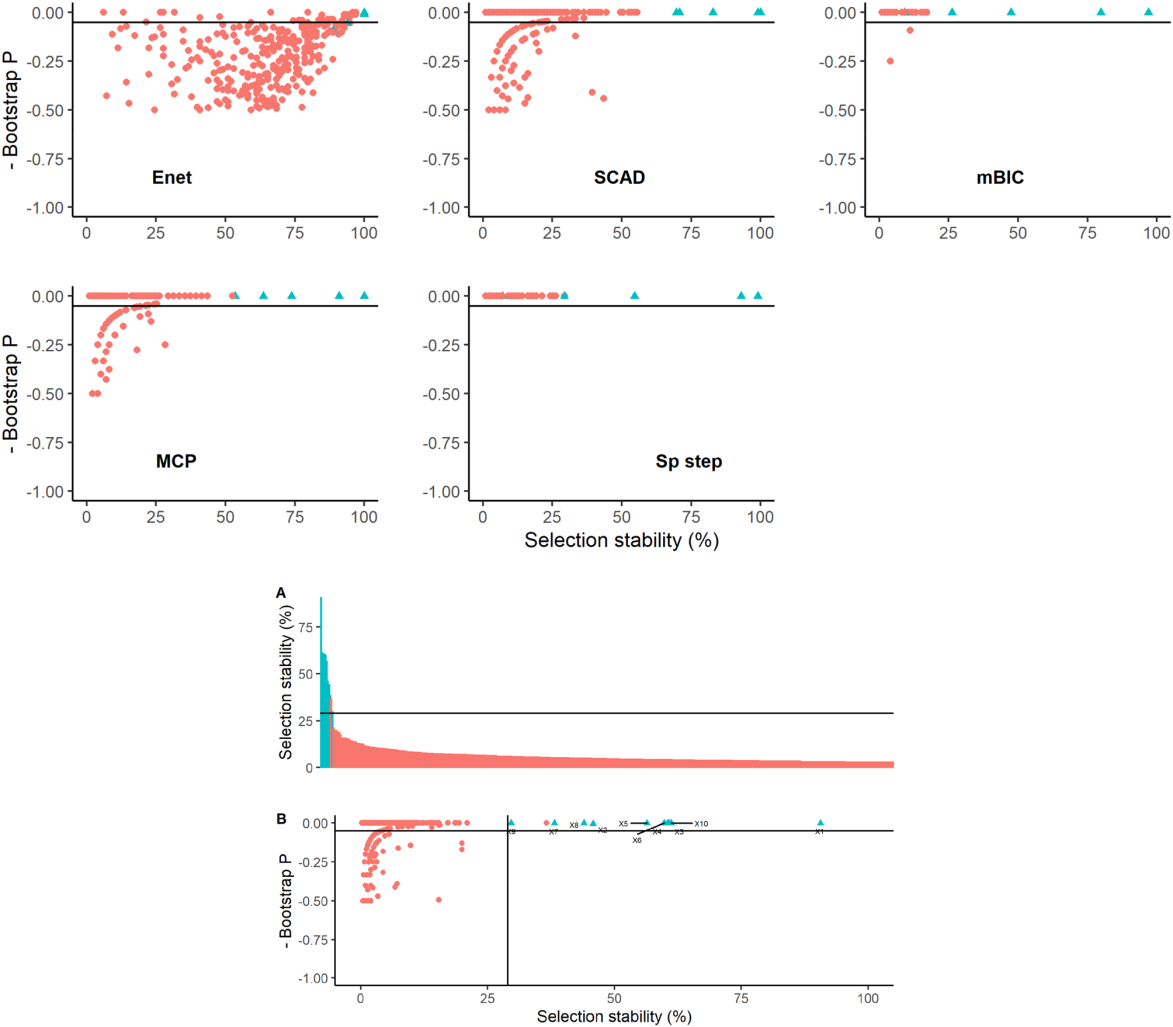
Graphical illustrations of bootstrap results from Dataset 4 using five methods of covariate selection. Graphs depict scatterplots of negative bootstrap P value against covariate selection stability except for Graph A that is an ordered plot of covariate selection stability in descending order for the combined model aggregating all five methods. The lines on graphs A and B represent the calculated threshold to determine a cut-off for ‘important’ covariates. The ‘true’ underlying covariates are coloured blue and labelled X1–X10.

Covariate selection stability and bootstrap P values for Datasets 3 and 4, are illustrated in Figs. 4 and 5 respectively. For Dataset 3, all methods tended to differentiate the true variables relatively well in terms of stability, with the MCP and SparseStep methods performing particularly well. The combined stability and bootstrap P values synthesised across methods for Dataset 3 are shown in Fig. 4. Again, the combined method provided a good separation of the true variables and using the rolling mean rate of change of selection stability > 1 to identify a threshold for ‘important’ variables the combined method resulted in a zero false positive and false negative error rates; demarcation of true variables was much clearer in this combined model than for the full models described in Table 3.

For Dataset 4, covariate stability and bootstrap P values are illustrated in Fig. 5. All methods differentiated the true variables to some extent in terms of stability, with again the MCP and SparseStep methods performing best. The combined stability and bootstrap P values calculated across all methods for Dataset 4 are also shown in Fig. 5. As with the stronger signal in Dataset 3, the combined method still provided a clearer separation of the true variables and using the rolling mean rate of change of selection stability > 1 to identify a threshold for ‘important’ variables, the combined method resulted in one false positive and no false negative covariates being identified, which was again markedly superior to the error rates of the full models (Table 3).

### Illustration of methods using a real dataset

Although in these data there were no known ‘gold standard’ covariates (i.e. those known to be causally associated with the outcome), we use a real field dataset to illustrate the concept of comparing and synthesising different methods of variable selection. The real data were gathered from a study conducted on 408 commercial sheep farms in the UK^16^ with a normally distributed outcome variable and 340 potential explanatory covariates.

**Figure 6:**
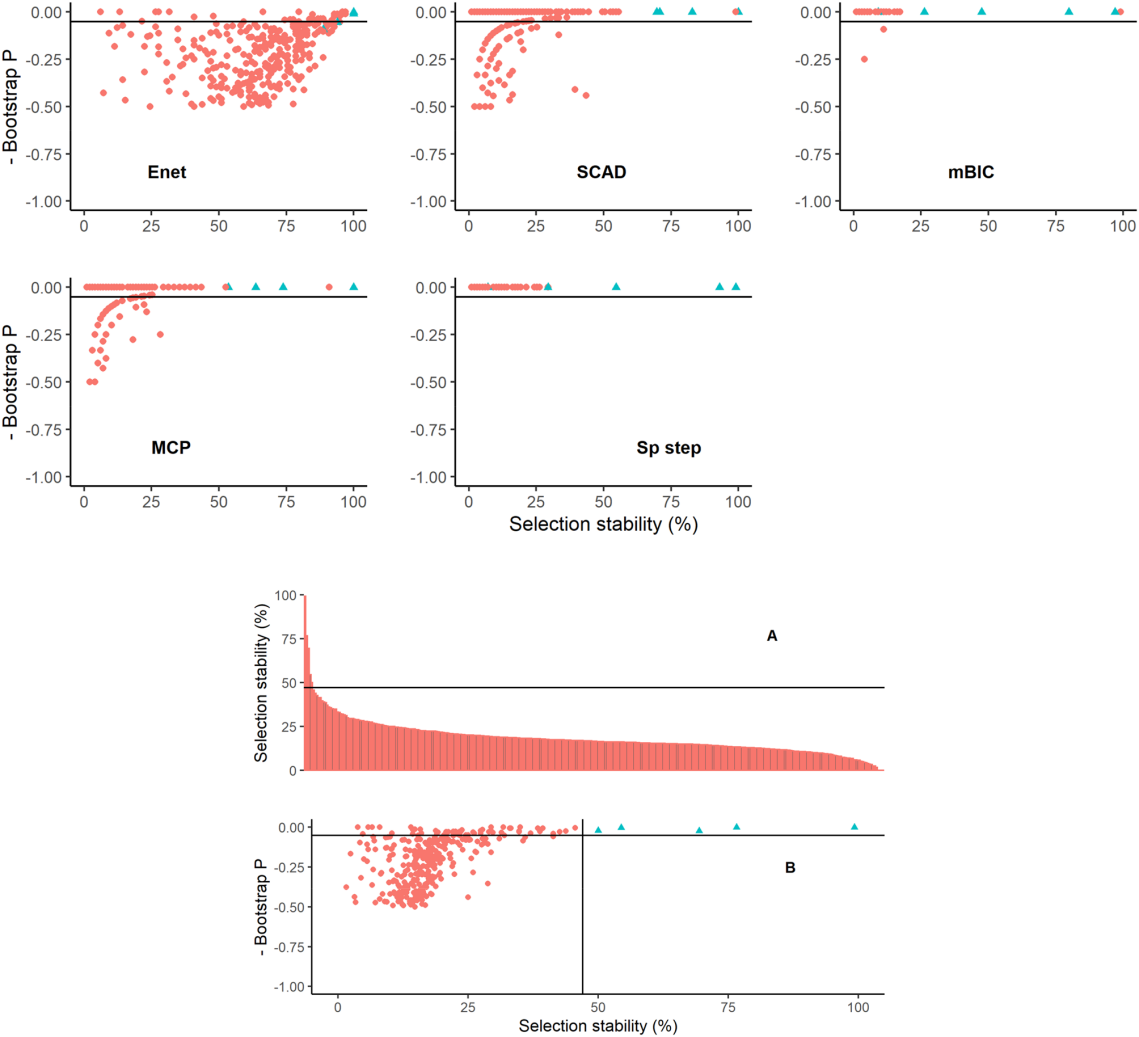
Graphical illustrations of bootstrap results from a real dataset using five methods of covariate selection. Graphs depict scatterplots of negative bootstrap P value against covariate selection stability except for Graph A that is an ordered plot of covariate selection stability in descending order for the combined model aggregating all five methods. The lines on graphs A and B represent the calculated threshold to determine a cut-off for ‘important’ covariates. The covariates marked in blue represent the five selected as most important in the combined method.

Covariate selection stability and bootstrap P values are displayed in Fig. 6 which allows visualisation of differences between method. SparseStep and mBIC produced the sparsest models such that few variables had a stability > 50% and in contrast, elastic net identified many variables with a stability > 50%, many of which also had a bootstrap P value < 0.05. The combination method, incorporating the threshold for selection stability, suggested that accounting for variability between methods, 5 covariates were identified as being most likely to be the most important of the 340 (Fig. 6). The individual method plots (Fig. 6) display where these 5 covariates were ranked by each individual method and illustrate how these become formally ranked when results of the methods are combined.

### Illustration of methods using datasets with no signal

To evaluate how the proposed combination method of covariate selection would perform when no important explanatory variables were present, we simulated two further datasets with no underlying signal. That is, an outcome variable was generated from a random normal distribution and potential explanatory covariates were randomly simulated independently of the outcome. Dataset 5 was comparable in size and structure to Datasets 1 and 2 with 910 potential explanatory variables, 1000 observations and correlations between explanatory covariates as described for Datasets 1 and 2. Dataset 6 was comparable in size and structure to Datasets 3 and 4 with 10,010 potential explanatory variables, 1000 observations and correlations between explanatory covariates as described for Datasets 3 and 4.

An identical analytic pathway was employed as described for Datasets 1–4 and illustrations of the resulting covariate selection stability and bootstrap P values are provided in Figs. S3 and S4 of Supplementary Information. It was noticeable that for both datasets, the maximum stability achieved by any variable for all models was generally lower than with Datasets 1–4. In addition, when using the combined method for both datasets, the selection stability did not exceed 50% for any covariate. Using the rolling mean rate of change of selection stability > 1 to identify a threshold for ‘important’ variables, in Dataset 5, the combined method resulted in a zero false positive covariates being identified and in Dataset 6, 2 false positive covariates were identified (FPER = 0.02%).

## Discussion

Despite the fact that many views have been expressed suggesting that analysis of individual datasets should be considered from multiple angles^12^ and that use of multiple analytic approaches may mitigate problems with scientific reproducibility^13,17^, it is rare that more than one technique is reported when conducting high-dimensional data analyses. In this paper we describe an approach to compare and combine results from different statistical methods used on one dataset, firstly to provide a basis to evaluate between-method variability and secondly to provide a means to formally combine and triangulate results between methods. Informal triangulation between statistical methods has been suggested as a route for researchers to confirm truly important variables^9^ but approaches to numerically combine results from different methods using the same data are lacking. With the approach proposed in this paper, triangulation is given a numeric foundation based on covariate stability and coefficient distributions. A graphical visualisation of selection stability and bootstrap P values was found useful to provide a framework to picture covariate importance of both individual and combined methods. In the combined method, covariates with highest stability and lowest bootstrap P values reflect those identified as most important overall by the individual methods and therefore those most sensible to be inferred of greatest importance in the data.

In these simulated datasets, the combined method generally performed as well or better than the individual methods; it tended to rank the true variables highly (in terms of stability and bootstrap P value) and gave coefficient estimates that, although slightly conservative (wider probability intervals than the true intervals), produced median values in close proximity to the true partial coefficient values. It should be noted, however, that the performance of the combined method will depend entirely on which individual methods are chosen to comprise the combination and will not necessarily produce an answer nearer to the truth than an individual method. Nonetheless, a comparison and combination of a variety of methods is still of use; it greatly adds to the transparency of analysis and helps ensure safety of results (by avoiding use of one specific ‘outlying’ method); this supports the view that use of multiple analytic approaches will lead to a better understanding of variability and important relationships within data^12,13,18^. Whilst it remains a truism that no models are right, but some can be useful^19^, we believe that using this comparison of individual and synthesised methods adds to the interpretation of high dimensional data analysis through transparently displaying differences between approaches and providing an overall result that incorporates this uncertainty.

Importantly, our results also confirm the recently highlighted issue that different analytic methods used on same data can yield different results^11^, both in terms of variables selected and coefficient estimates^9^. The simulated datasets used in this study, in which the true underlying relationships were known, were useful to illustrate such differences between methods. Differences between methods occurred in sparsity of solution, the magnitude and relative ranking of variable stability and coefficient estimates. Clear differences were also identified between methods in the analysis of datasets that contained no signal (Supplementary Information Figs. S3 and S4). Such variability confirms the value of assessing data from multiple angles. Indeed it was notable that in the simulated datasets with a weaker signal (i.e. with more random variation from unknown true variables included to calculate the outcome), the variability of results between methods was greater than when a strong signal was present (and the combined method was more valuable in terms of selecting the true variables), suggesting that in these circumstances, use of multiple methods may be even more important. Since when collecting high dimensional research data we cannot depend on necessarily having strong signals from explanatory variables, the use of multiple methods appears to be a pragmatic solution.

Although the use of selection stability is recognised to facilitate robust solutions in statistical modelling^14,15,20^ and was found to be useful to clarify covariate selection in this study, there remains an issue of how to determine an exact threshold of stability at which a variable is deemed ‘important’. However, graphical representation of variable stability in descending order indicated, in this study, a region in which stability tended to change from being relatively high to relatively low; in data with relatively sparse solutions, this is likely to be the case. It has been shown that the chosen threshold selection probability alongside the number of possible explanatory covariates determines the rate of false positive covariates selected^14^ and this can be seen for all methods used in this study; as the stability threshold increases, the false positive rate decreases. However, rather than concentrating on an exact threshold value, we believe covariate stability can be viewed more as a continuum to rank variables according to ‘likelihood of true positivity’. Since the higher the stability, the less likely a covariate is to be a false positive^14,21^, a relatively high threshold could be chosen in studies in which false positive variables need to be avoided. However, not all studies are of this nature and, for example, when screening for potentially important explanatory variables for follow up intervention studies, it may be more prudent to avoid false negative covariates and hence a lower stability threshold could be considered. The real field dataset used in this study illustrated this point (Fig. 6) and a threshold stability between 40 and 60% could be used depending on the requirement to either minimise false positive or false negative findings in that particular study.

For the simulated data used in this study we employed a sparsity assumption; the outcome variable was calculated to depend on a relatively small number of variables in the data. Such an assumption is commonly used when evaluating high dimensional data^4^ but it should be noted that our results relate to this circumstance. Additional research comparing the performance of statistical methods with less sparse solutions would be of interest.

Similarly, although these principles of model comparison and combination of results could be applied to any suite of methods, our results apply to the specific methods we chose. Our choice was based on methods that were reasonably common and with literature to support their validity^5,6,8,22^, but the choice was arbitrary. A greater understanding would be useful of which statistical methods are best to combine in different circumstances, but despite this, a comparison and combination of several methods when analysing an individual dataset is likely to be fruitful to evaluate the extent of between-method variation and to triangulate results. Further insights are needed on the applicability of method synthesis for different model types (e.g. those including random effects, non-linearities or with categorical outcomes), although it is likely the same principles will apply. Furthermore, additional research would be useful to develop methods to determine the optimal threshold for selection stability, to minimise error rates for any given dataset and model.

In conclusion, in this paper we have developed an adaptable, straightforward method to compare and synthesise results across any chosen number of variable selection techniques to formally triangulate results between methods. Importantly, the method includes selection stability as well as coefficient estimates and provides a unified result that incorporates uncertainty arising between methods.

## Materials and methods

To evaluate and combine results from six methods of covariate selection, six simulated datasets containing known relationships were generated. Initially, two datasets were constructed, these are described in detail below. Subsequently, two larger datasets were constructed using similar methods and an overview of these is also provided. Finally, the combination of variable selection techniques were evaluated using one real dataset and two datasets containing no signal (i.e. comprising variables generated at random); these datasets are also briefly described.

### Data simulation

The purpose was to construct realistic datasets in which a ‘true’ set of predictor variables were known and from which an outcome variable was directly calculated. Simulated datasets 1 and 2 both contained 1000 rows and 910 columns. Ten variables were set as being ‘causal’ and used to directly calculate an outcome variable, y. These variables were simulated from the following distributions;

Variables X1–X5 were drawn from a multivariate normal (using mvrnorm function in the MASS package in R^23^), each variable drawn from a distribution with mean = 0, SD = 1 and covariance matrix specified such that variables were drawn with a correlation between each of 0.6. The purpose of these correlations was to mimic reality; causal variables are often correlated in epidemiological data.

Variables X6-X10 were drawn independently from a random normal distribution with mean = 0 and SD = 1.

An outcome variable, y, was generated from these 10 variables as follows;

1 $$y=Intercept+ \sum_{j=1}^{t} 2.5{x}_{j}+{x}_{k}+\sum_{l=1}^{s} 2.5{x}_{l}+v$$

where $Intercept$ =1, ${x}_{j}$ represented the jth of $t$=4 of the correlated covariates, ${x}_{k}$ was the additional correlated variable, ${x}_{l}$ represented the lth of $s$=5 of the uncorrelated covariates and *v*, a random variable that represented all other real but unknown effects that causally influence y, was drawn from a Normal distribution with mean = 0 and standard deviation which varied in the two datasets as follows;

- Dataset 1: *v* = N(0,15).
- Dataset 2: *v* = N(0,6.5).

The ten variables used to calculate y, we refer to as the ‘true’ explanatory covariates throughout the manuscript. For each dataset a ‘true’ model was estimated using conventional linear regression in R^24^ using solely these ten simulated variables. The coefficient distributions from these models were taken as the ‘true’ underlying distributions for comparison in subsequent analysis. The true coefficients for the covariates derived from Datasets 1 and 2 are provided in Table 2 in the “Results” section.

An additional 900 ‘noise’ variables were simulated from distributions with mean = 0 and SD = 1; these had no dependence or relation to the outcome variable y, i.e. they were drawn from independent random distributions. These were deemed ‘false’ variables, not causally related to y. Since in epidemiological data it is common that such non-causal variables may also contain correlations, 400 of the 900 variables were drawn from multivariate Normal distributions as follows. Four sets of 50 variables were drawn from a MVN distribution such that each variable was drawn with a mean = 0 and SD = 1 and the correlation between individual variables within sets of 50 variables was 0.7. Therefore, this resulted in four sets of 50 variables correlated at ~ 0.7. This procedure was repeated for another four sets of 50 variables but for these the correlation was 0.8. A final group of 500 variables were drawn independently from random Normal distributions with mean = 0 and SD = 1.

Therefore, the resulting simulated dataset contained an outcome variable that was calculated from ten ‘true’ variables (with an additional random term included to reflect unknown but causal influences on the outcome) and an additional 900 ‘false’ (noise) variables, simulated at random and independent of the outcome.

Two further simulated datasets were generated, Datasets 3 and 4, using the same principles but of different dimensions; with 200 rows and 10,010 columns (potential explanatory variables). The datasets again contained 10 ‘true’ variables that were simulated as described for Datasets 1 and 2 (Eq. (2)), except the following random terms were used to reflect additional unknown but casual effects;

- Dataset 3: *v* = N(0, 6.5).
- Dataset 4: *v* = N(0, 23).

As with datasets 1 and 2, a ‘true’ model was estimated using conventional linear regression from these ten simulated variables and coefficient distributions from these models are illustrated in Figs. S1 and S2 in Supplementary Materials. For Datasets 3 and 4, an additional 10,000 variables were simulated from random Normal distributions with mean = 0 and SD = 1. As for Datasets 1 and 2, a proportion of these variables were drawn from multivariate normal distributions as follows. Forty sets of 50 variables were drawn from a MVN distribution such that each variable was drawn with a mean = 0 and SD = 1 and the correlation between variables of 0.7. A further 40 sets of 50 variables were drawn from multivariate random normal distributions with mean = 0 and SD = 1 and the correlation between variables of 0.8. A final 6000 variables were drawn independently at random from Normal distributions with mean = 0 and SD = 1. Therefore, Datasets 3 and 4 contained 200 rows and 10,010 columns of which 10 were ‘true’ variables. The rationale for using Datasets 3 and 4 was to evaluate the effectiveness of covariate selection methods in data where the number of predictors (p) was far greater than the number of observations (n).

Datasets 5 and 6 comprised 1000 rows by 910 covariates and 200 rows by 10,000 covariates respectively, but an outcome variable was generated at random (i.e. independent of and with no underlying relationship to, the potential explanatory covariates).

Finally a set of field data were used to illustrate the proposed modelling approaches. This real dataset was gathered from 408 commercial farms in the UK^16^, had an outcome variable that was approximately normally distributed and a set of 340 potential explanatory variables available for selection. Explanatory variables were standardised for analysis; details of the data, pre-processing and variable descriptions have been reported previously^16^.

### Models used for variable selection

To evaluate the effectiveness of retrieval of the ten ‘true’ variables from within the simulated datasets, six methods were employed and compared. These were; a conventional stepwise selection method based on AIC (stepwise linear regression; SLR)^25^, elastic net regression (Enet)^8^; smoothly clipped absolute deviation (SCAD)^6,26^, minimax convex penalty (MCP)^27^, Sparsestep (S_step)^22^ and a modified Bayesian Information Criterion (mBIC)^5^. The basis for model selection for each of these methods is outlined below.

*Stepwise linear regression based on AIC (SLR).*

A conventional linear regression model was implemented using the ‘stepwise’ function in the Bigstep package^28^ in R^24^. The regression equation took the form;

2 $$y={\beta }_{0}+ \sum_{j=1}^{p}{ \beta }_{j}{x}_{j} +e$$

where $y$ was the response variable specified in the simulated data, ${\beta }_{0}$ an intercept term, ${x}_{j}$ represented the jth of $p$ covariates with an estimated coefficient ${\beta }_{j}$, $e$ was the residual model error. For computational reasons, covariate selection was conducted by first removing explanatory variables with a relatively poor correlation with the outcome (P > 0.80) followed by a stepwise procedure with selection of variables achieved through minimisation of the Akaike information criterion (AIC) as the loss function (AIC defined as 2$\mathrm{k}-2\mathrm{ln}(\widehat{\mathrm{L}})$ where k is the number of parameters in the model and $\widehat{\mathrm{L}}$ the likelihood function). Therefore the number of covariates (p) selected in the final model was determined by the AIC loss function.

#### Elastic net regression

Elastic net is a form of regularised regression that incorporates a mixture of the lasso (L1) and ridge (L2) penalties and can be described as;

3 $$  SSE_{{enet}}  = \frac{1}{{2n}}~\mathop \sum \limits_{{i = 1}}^{n} \left( {y_{i}  - {\hat{y}}_{i} } \right)^{2}  + {\lambda _{E}}   \left[ {\mathop \sum \limits_{{j = 1}}^{P}  {\frac{1}{{2}}\left( {1 - \alpha } \right) \beta _{j}^{2} } + \alpha \left| {\beta _{j} } \right|} \right] $$

where $SS{E}_{enet}$ represente the elastic net loss function to be minimised, i denoted each simulated observation and n the number of observations, y_i_ and ŷ_i_ were respectively the simulated and model predicted outcome for the ith observation, j denoted a predictor variable with p the number of predictor variables selected in the model through minimisation of the loss function (some variable coefficients are set to zero meaning the

y are not selected in a final model), and |β| represented absolute values of the regression coefficients. The hyperparameters that represent the penalty (λ_E_) and the relative proportion of penalisation on either the sum of the square of the coefficients or the unsquared coefficients (α) were optimised by 10 × tenfold cross validation to minimise mean absolute error (MAE).

Elastic net models were built using the glmnet package^29^ using the caret package platform^30^ in R^24^.

*Smoothly clipped absolute deviation and minimax convex penalty.*

Smoothly clipped absolute deviation (SCAD)^26^ and minimax convex penalty (MCP)^27^ are also forms of regularised regression. A feature of these methods is that the size of the penalty function varies with the size of variable coefficient, β. Both methods can be described by the general framework;

4 $$SSE_{{scad/mcp}} = \mathop \sum \limits_{{i = 1}}^{n} \left( {y_{i} - \hat{y}_{i} } \right)^{2} + \mathop \sum \limits_{{j = 1}}^{p} P~(\beta _{j} |\lambda ,\gamma )$$

where SSE_scad/mcp_ represents the SCAD or MCP loss function to be minimised, i, y_i_, ŷ_i_, j, p and n are as defined in Eq. (3) and $P\left({\beta }_{j}\right|{\varvec{\lambda}},\gamma )$ represents a penalty function as follows;

For SCAD:

5 $$P{(}\beta {{|\lambda }},\gamma ) = \begin{array}{*{20}l} {{\lambda },} \hfill & {{\text{if }}\;\;\left| {\upbeta } \right| \le {\lambda }} \hfill \\ {\frac{{\gamma {\lambda } - { }\left| {\upbeta } \right|}}{\gamma - 1}} \hfill & {{\text{if}}\;{{ \lambda }}\left\langle {\left| {\upbeta } \right|} \right\rangle \gamma {\lambda }} \hfill \\ {0,} \hfill & {{\text{if}}\;{ }\left| {\upbeta } \right| \ge \gamma {\lambda }} \hfill \\ \end{array}$$

For MCP:

6 $$P{(}\beta {{|\lambda }},\gamma ) = \begin{array}{*{20}l} { {\lambda }\left| {\upbeta } \right| - \frac{{\beta^{2} }}{2\gamma },} & {{\text{if}}\;{ }\left| {\upbeta } \right| \le \gamma {\lambda }} \\ {0.5\gamma {\lambda }^{2} } & { {\text{if}}\;{ }\left| {\upbeta } \right| > \gamma {\lambda }} \\ \end{array}$$

where ${\varvec{\upgamma}}{\text{ and }}{\varvec{\uplambda}}$ are hyperparameters optimised using 10 × tenfold cross validation to minimise the MAE. Both SCAD and MCP models were estimated using the R package ncvreg^31^.

#### SparseStep

The SparseStep function provides another approach for non-linear penalisation in the regression loss function^22^. The SparseStep loss function can be described as;

7 $$SSE_{sp\_step} = \mathop \sum \limits_{i = 1}^{n} \left( {y_{i} - \hat{y}_{{\text{i}}} } \right)^{2} + {\lambda }\mathop \sum \limits_{j = 1}^{p} \frac{{\beta^{2} }}{{\beta^{2} + \gamma^{2} }}$$

where SSE_sp_step_ represents the SparseStep loss function to be minimised, i, y_i_, ŷ_i_, j, p and n are as defined in Eq. (3) and $\lambda \mathrm{and} \gamma$ are hyperparameters optimised using 10 × tenfold cross validation to minimise the MAE. The Sparsestep model was estimated using the sparsestep package in R^22^.

#### Modified Bayesian Information Criterion (mBIC)

A modified Bayesian Information Criterion was used for model selection implemented in the R package bigstep^28^. This modification of the BIC effectively increases the penalty on the number of parameters selected in the model beyond the conventional BIC, hence producing a sparser model. The loss function on which stepwise variable selection is based^5^ can be represented as;

8 $$mBIC = logL(Y|M_{i,} \theta_{i} ) - \frac{1}{2 }k_{i} logn - k_{i } {\text{log}}\left( {\frac{1 - pr}{{pr}}} \right)$$

where $logL(Y|{M}_{i,}{\theta }_{i})$ represents the log likelihood given model ${M}_{i}$ and parameter values ${\theta }_{i}$, k is the number of predictors in the selected model, n the sample size, and *pr* the probability that a randomly chosen predictor influences Y. As the number of available predictors increases relative to the number of samples (n), *pr* decreases and ${{\varvec{k}}}_{{\varvec{i}}\boldsymbol{ }}\mathrm{log}(\frac{1-pr}{pr})$ becomes of increasing importance as a penalty term.

### Estimation of selection stability and coefficient distributions

Conventional bootstrapping^32^ was used to estimate covariate stability for all analytic approaches, according to methods previously described^9^. In brief, selection stability^14,15,20^ was evaluated for each model as the percentage of times that each covariate was selected in the model across bootstrap samples. The distributions of variable coefficients were calculated from all non-zero values of the coefficient in the bootstrap samples; this allowed comparisons between methods of variable selection stability as well as coefficient estimates. A further evaluation of the importance of each variable in the final bootstrapped model was made from all non-zero values (i.e. when the variable was selected in the model), by estimating what we term the Bootstrap P value. The Bootstrap P value was calculated as the smallest proportion of (non-zero) coefficient values to one side of zero. That is, if a covariate was selected in the model on 80 occasions (i.e. 80 non zero values) and 70 of these were either greater or less than zero, then the Bootstrap P value would be (80–70)/80 = 0.125. For all methods, variable selection and importance were visualised by plotting selection stability against Bootstrap P value for all variables in the dataset.

### Synthesis of results across methods

Coefficient distributions and stabilities were synthesised across methods (with the exception of Stepwise Linear Regression based on AIC which performed poorly) by aggregating the bootstrap matrix by row from each individual method; matrices were aligned by variable. Therefore, an enlarged ‘combined’ matrix containing an equal number of bootstrap samples for each method was produced comprising variables and coefficient estimates aggregated across all methods; this we term the combined model. Since an equal number of bootstrap samples were used for each method (n = 500 for Datasets 1 and 2, n = 100 for all other datasets, to speed computation), an equal weighting was given to each method when calculating synthesised coefficient estimates and Bootstrap P values. From this combined matrix, an overall selection stability and Bootstrap P value were calculated as described above. Coefficient distributions for each covariate were also calculated from all non-zero values of the combined matrix. Therefore the combined model resulted in estimates for selection stability, coefficient distributions and Bootstrap P values derived for all covariates across all five methods of covariate selection.

### Evaluation and comparisons of model performance

Model performance was assessed using false positive and false negative rates (FPER and FNER respectively). For each model, these were defined as;

- FPER = Number of false positive covariates selected/Total number true negative covariates in data.
- FNER = Number true positive covariates not selected/Total number true positive covariates in data.

For bootstrapped models, since both stability and Bootstrap P value were key for evaluating variable importance, we visualised and compared these parameters graphically. For the combined method, covariates were ranked and plotted in order of descending stability to identify the region where stability changed from being relatively high to low; the same principle as used in a classical scree plot. To evaluate stability objectively in the combined method, the rate of change in stability between consecutive covariates ranked in descending order was determined and a smoothed mean calculated for the rate of change in stability using 15 consecutive values. A sensitivity analysis was conducted on the number of values to include in calculation of the smoothed mean and for values between 10 and 20, there were negligible differences in results. A value under 10 gave insufficient smoothing and over 20 started to show a lack of discrimination, therefore a value of 15 was chosen. Once the rolling mean rate of change reached a value ≤ 1 (i.e. the rate of change moved from being > 1 to ≤ 1), this was deemed to signify the change point in stability for that model and used as a threshold to calculate FPER and FNER (see Fig. S5, Supplementary Information).

## Supplementary Information

Supplementary Information.
