# Supplementary material for: Model selection for inferential models with high dimensional data: synthesis and graphical representation of multiple techniques
Source: Sci Rep. 2021 Jan 11;11:412. doi: 10.1038/s41598-020-79317-8 (PMC7801732; doi:10.1038/s41598-020-79317-8)
Supplement: Supplementary file 1 — Supplementary Information. [file 41598_2020_79317_MOESM1_ESM.docx]

**Model selection for inferential models with high dimensional data: Synthesis and graphical representation of multiple techniques**

**Author names and affiliations**

Eliana Lima^a^, Robert Hyde^a^, Martin Green^a^

^a^ School of Veterinary Medicine and Science, University of Nottingham, Sutton Bonington Campus, Leicestershire, United Kingdom

**Corresponding author**

Martin Green

E-mail: martin.green@nottingham.ac.uk

School of Veterinary Medicine and Science, University of Nottingham, Sutton Bonington Campus, Leicestershire, LE12 5RD, United Kingdom

Tel +44 115 951 6116

## **Supplementary Information**

**
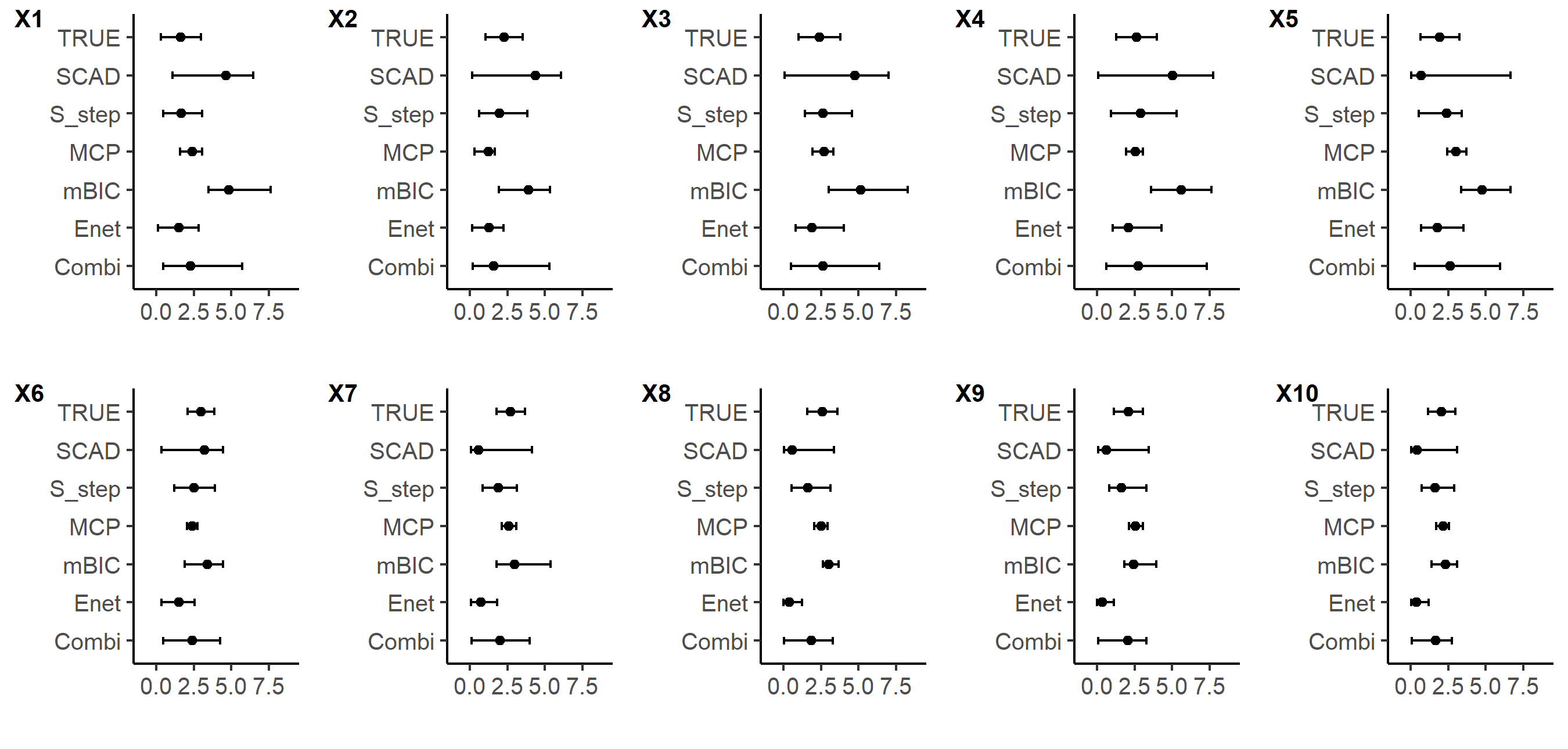
**

Key; X-axes – covariate coefficient value, X1 to X10 – covariate names, TRUE – the correct partial coefficient for each covariate, SCAD - smoothly clipped absolute deviation, S_step – SparseStep regression, MCP - minimax convex penalty, mBIC - modified Bayesian Information Criterion, Enet – elastic net regression, Combi – Combined method; results aggregated from all five techniques.

**Figure S1. *Bootstrap coefficient distributions of the true covariates in Dataset 3, estimated using five different statistical techniques and a combination method incorporating all five. ‘TRUE’ represents the actual underlying target partial coefficients.***

**
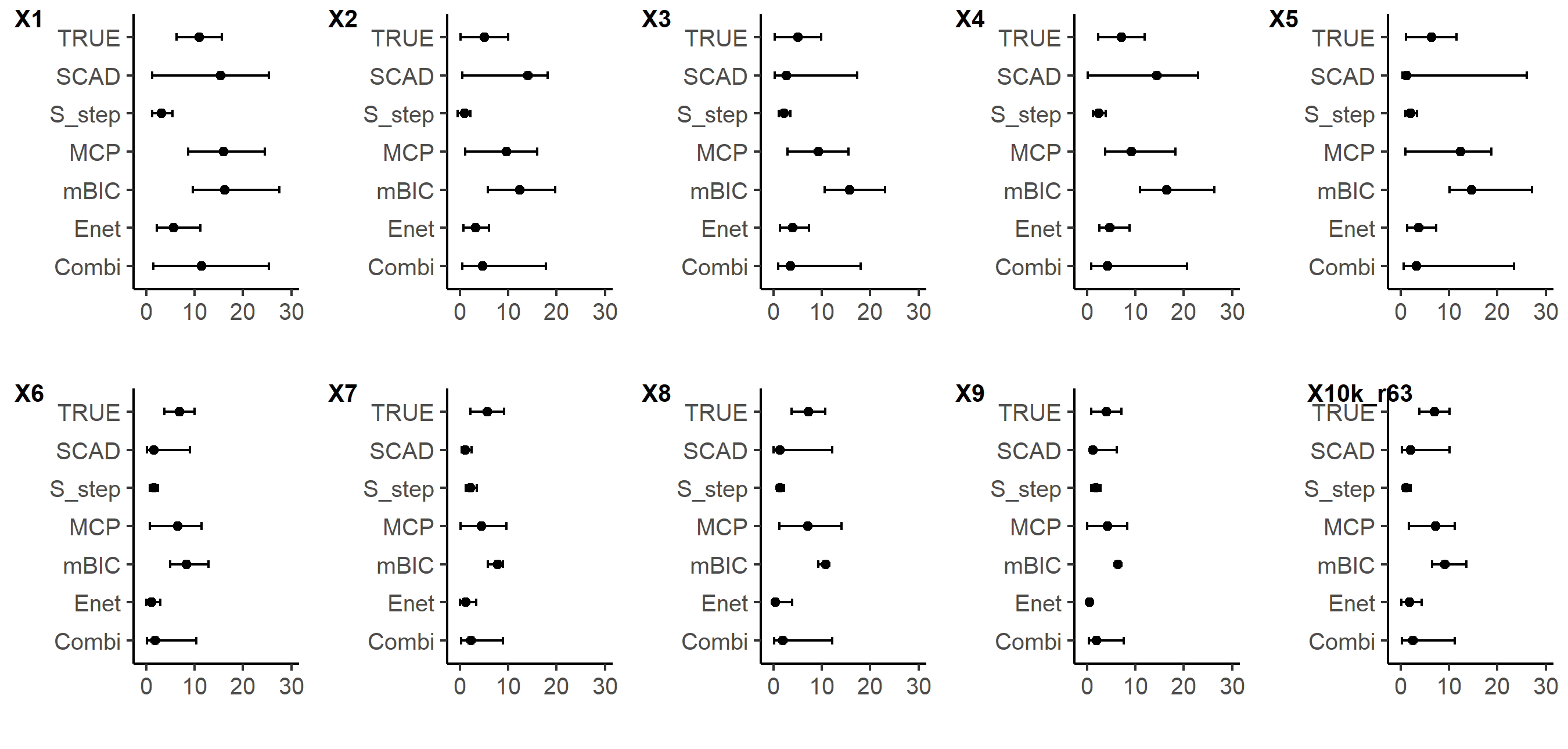
**

Key; X-axes – covariate coefficient value, X1 to X10 – covariate names, TRUE – the correct partial coefficient for each covariate, SCAD - smoothly clipped absolute deviation, S_step – SparseStep regression, MCP - minimax convex penalty, mBIC - modified Bayesian Information Criterion, Enet – elastic net regression, Combi – Combined method; results aggregated from all five techniques.

**Figure S2. *Bootstrap coefficient distributions of the true covariates in Dataset 4, estimated using five different statistical techniques and a combination method incorporating all five. ‘TRUE’ represents the actual underlying true partial coefficients.***

**
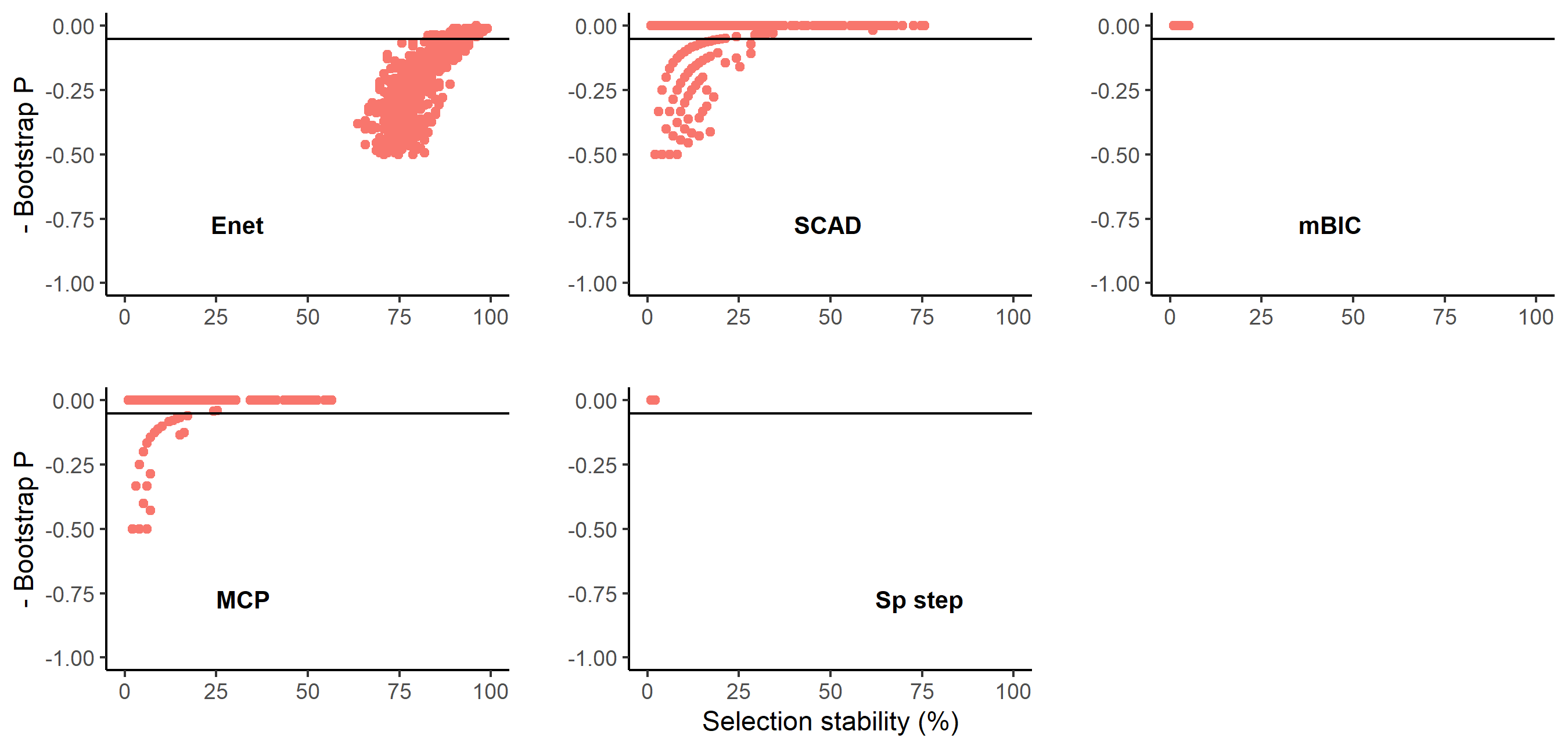
**

**
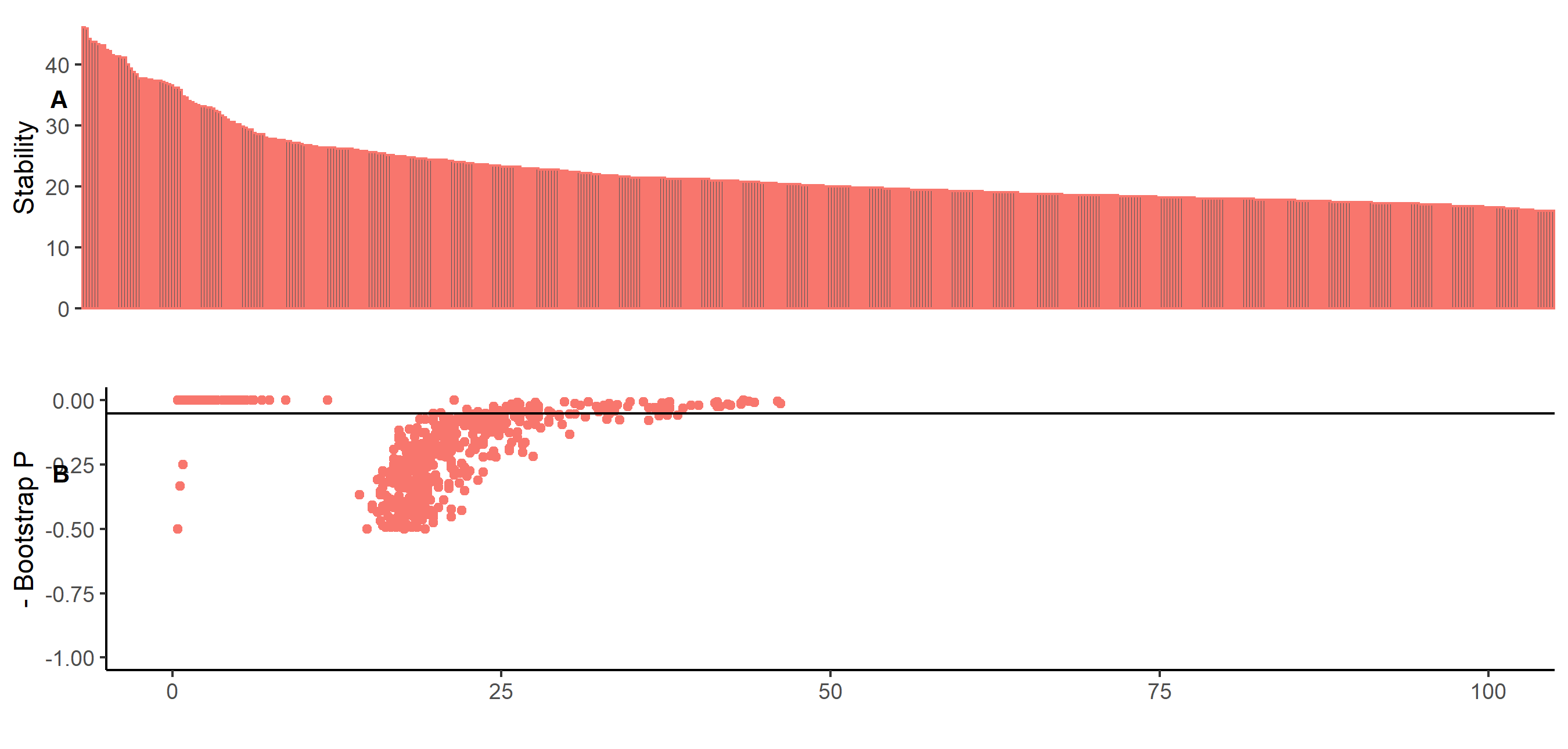
**

Key; Enet – elastic net regression, SCAD - smoothly clipped absolute deviation, mBIC - modified Bayesian Information Criterion, MCP minimax convex penalty, Sp step – SparseStep regression, A and B - Combined method with results aggregated from all five techniques

**Figure S3. *Graphical illustrations of bootstrap results from Dataset 5 (contained no ‘true’ covariates) using five methods of covariate selection. Graphs depict scatterplots of negative bootstrap P value against covariate selection stability except for Graph B that is an ordered plot of covariate selection stability in descending order for the combined model aggregating all five methods. All covariates fell below a calculated threshold to determine a cut-off for being ‘important’.***

**
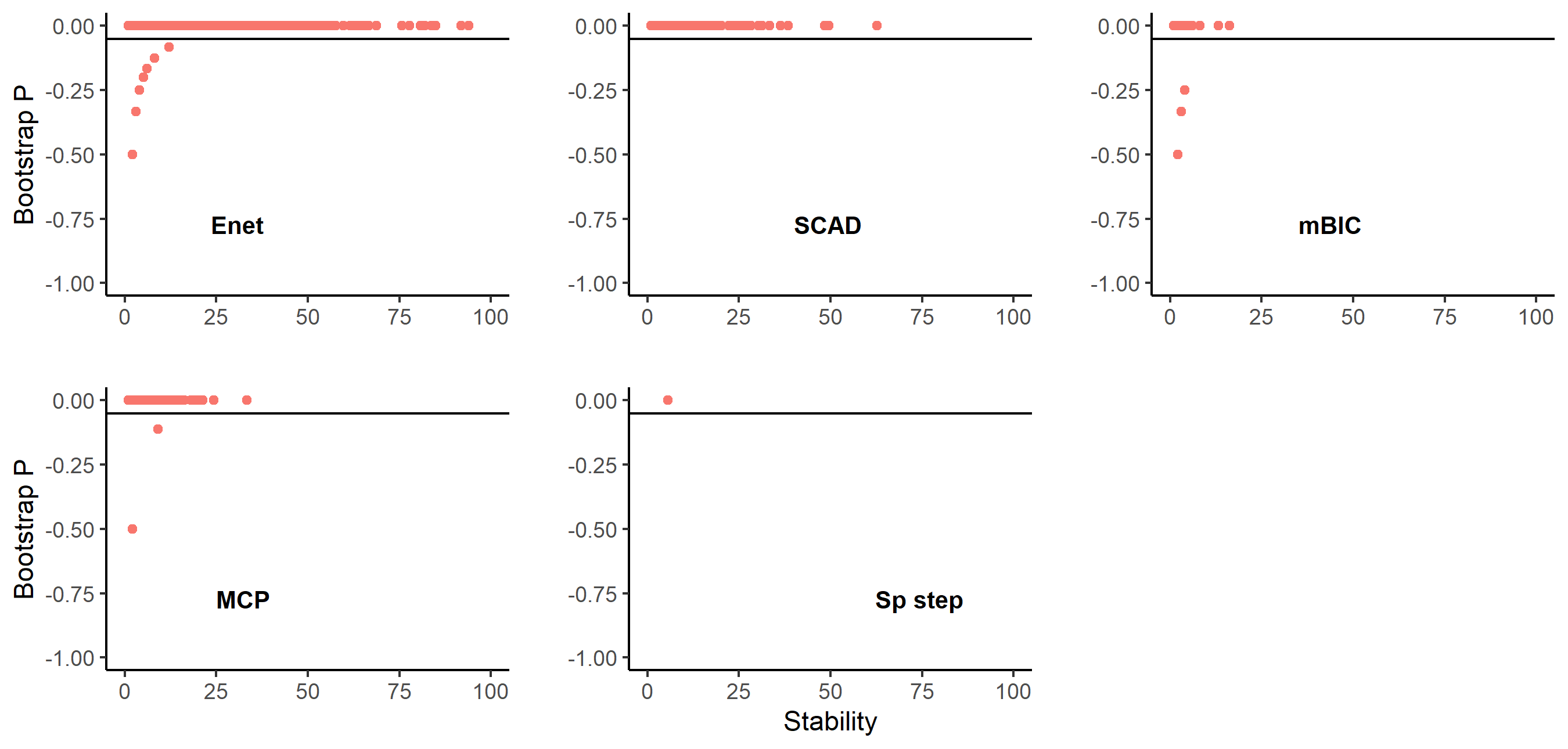
**

**
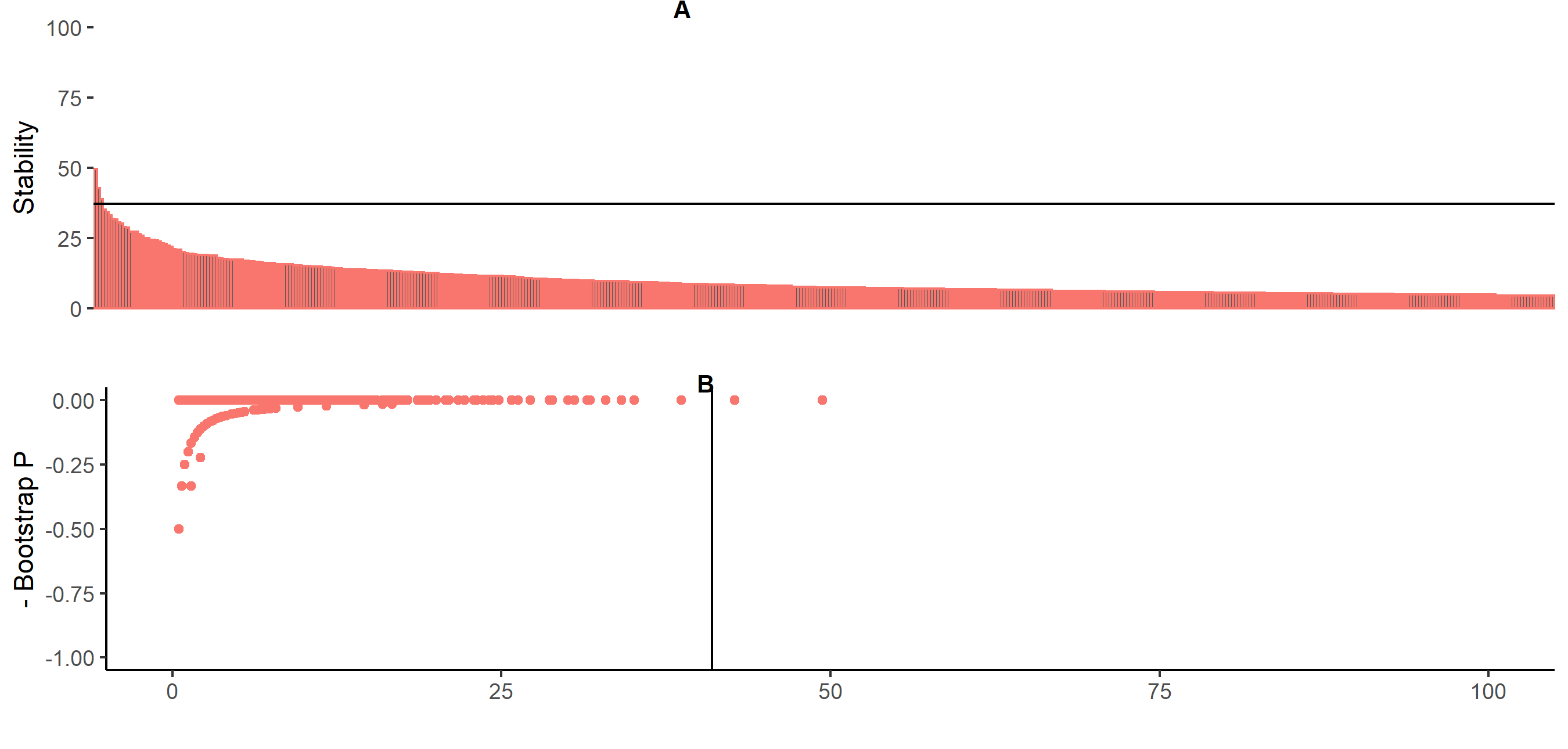
**

Key; Enet – elastic net regression, SCAD - smoothly clipped absolute deviation, mBIC - modified Bayesian Information Criterion, MCP minimax convex penalty, Sp step – SparseStep regression, A and B - Combined method with results aggregated from all five techniques

**Figure S4. *Graphical illustrations of bootstrap results from Dataset 6 (contained no ‘true’ covariates) using five methods of covariate selection. Graphs depict scatterplots of negative bootstrap P value against covariate selection stability except for Graph B that is an ordered plot of covariate selection stability in descending order for the combined model aggregating all five methods. The lines on graphs A and B represent the calculated threshold to determine a cut-off for ‘important’ covariates; two covariates were identified as false positives (FPER = 0.02%).***

**Rate of change of stability plots to determine threshold**

These are based on a smoothed mean change in selection stability (ΔS) between consecutive stability values (calculated from 15 consecutive values for smoothing) having ordered variables in descending order of stability. The rationale is that once the mean rate of change in a region = 1 then the tangent of the curve in that region is 45 degrees and this signifies the ‘change point’ in the rate of change of stability.

**
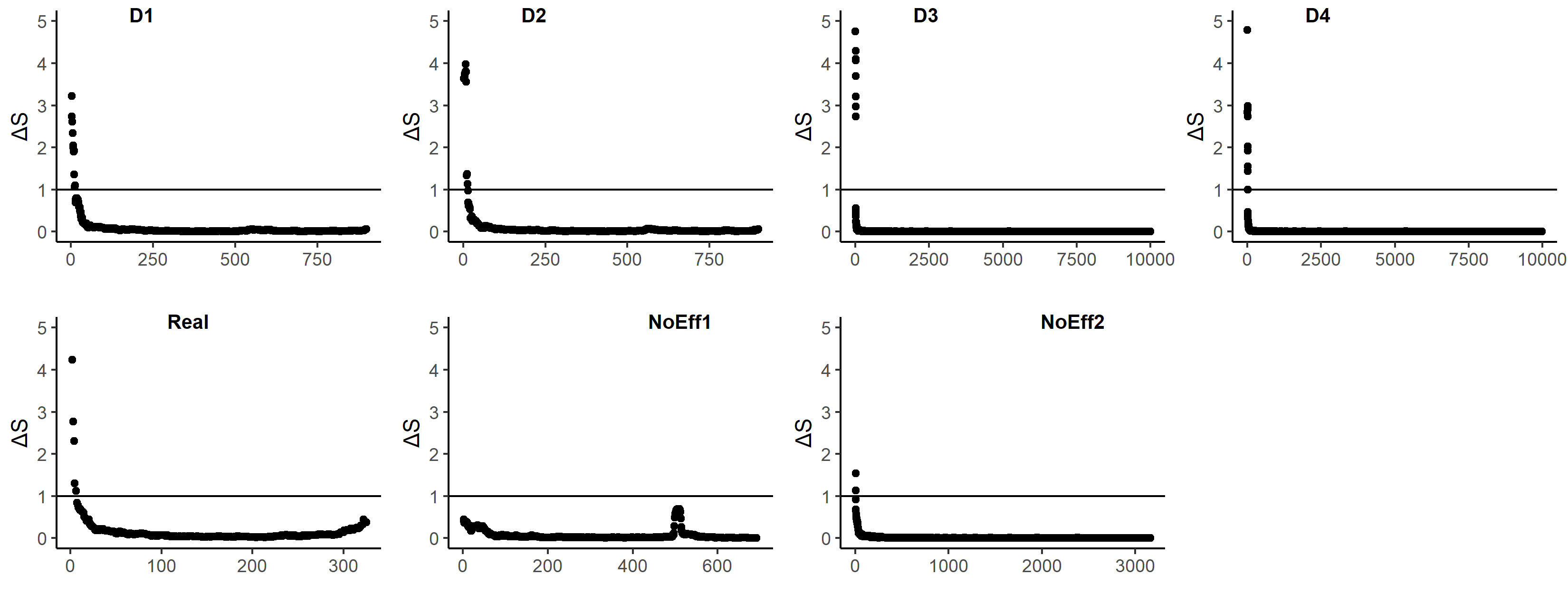
**

Key; D1 to D4 – Simulated datasets 1-4, Real – The Real Datastet, NoEff1 – Simulated Dataset 5 with no true covariates, NoEff2 – Simulated Dataset 6 with no true covariates.

***Figure S5. Graphs for the combined model used on seven different datasets to illustrate the smoothed mean rate of change in selection stability (ΔS) between consecutive covariates ranked in descending order of stability. The horizontal lines on each graph identify ΔS = 1, the point used as a threshold to calculate error rates.***
